# Expression of TSG101 protein and LSF transcription factor in HPV-positive cervical cancer cells

**DOI:** 10.3892/ol.2014.1967

**Published:** 2014-03-11

**Authors:** JUSTYNA K. BRONIARCZYK, ALICJA WAROWICKA, ANNA KWAŚNIEWSKA, MARIA WOHUŃ-CHOLEWA, WOJCIECH KWAŚNIEWSKI, ANNA GOŹDZICKA-JÓZEFIAK

**Affiliations:** 1 Department of Molecular Virology, Adam Mickiewicz University, Poznań 61-614, Poland; 2 NanoBioMedical Centre, Adam Mickiewicz University, Poznań 61-614, Poland; 3 Department of Obstetrics and Gynecology, Medical University of Lublin, Lublin 20-081, Poland; 4 Department of Cell Biology, University of Medical Science, Poznan 60-806, Poland; 5 First Department of Oncological Gynecology and Gynecology, Medical University of Lublin, Lublin 20-081, Poland

**Keywords:** LSF transcription factor, TSG101 protein, cervical cancer

## Abstract

Our previous study demonstrated a decreased expression of tumor susceptibility gene 101 (*TSG101*) in cervical cancer cells. To identify the mechanism responsible for TSG101 downregulation during cervical cancer development, we analyzed the TSG101 promoter using cis-element cluster finder software. One of the transcription factors whose binding site was detected in the *TSG101* promoter was late SV40 factor (LSF). The aim of this study was to analyze the TSG101 protein and LSF expression levels during cervical cancer development. Immunohistochemical analysis confirmed a previously observed decreased expression of TSG101, whereas quantitative polymerase chain reaction (qPCR) and immunohistochemistry analysis revealed high expression of LSF in cervical, precancer and cancer cells compared with human papillomavirus (HPV)-negative non-cancer samples. High expression of LSF in cervical cancer HPV-positive cells suggests that this protein may be important in the regulation of TSG101 expression, as well as in cervical carcinogenesis. The role of LSF as a mediator in cervical cancer development must be confirmed in future studies.

## Introduction

Human papillomavirus (HPV)-mediated transformation of human cervical epithelial cells has been recognized as a multi-step process in which not only viruses but also certain additional unknown factors and (epi)genetic events are required.

Our previous study indicated that one of the factors that may be involved in cervical carcinogenesis is tumor susceptibility gene 101 (*TSG101*) (1). The *TSG101* gene was mapped to chromosome 11.p15.1–p15.2., a region that is associated with the loss of heterozygosity in several tumor types, including breast cancer and cervical cancer (2,3). The TSG101 protein is involved in a variety of important biological functions, such as ubiquitination, transcriptional regulation, endosomal trafficking, virus budding, proliferation and cell survival (4–16). It has been suggested that TSG101 is an important factor for maintaining cellular homeostasis and that perturbation of TSG101 functions leads to transformation (17). To identify the mechanism responsible for TSG101 downregulation during cervical cancer development, we analyzed the *TSG101* promoter using cis-element cluster finder (Cister) software. One of the transcription factors, whose binding site was detected in the *TSG101* promoter, was LSF (1).

LSF was initially identified due to its ability to activate a major late Simian virus 40 (SV40) promoter (18). Late SV40 factor (LSF) is commonly named as CP2 and is encoded by the *TFCP2* gene located in chromosome 12q13 (19,20). LSF belongs to an evolutionary conserved family of transcription factors consisting of two subfamilies: LSF/CP2 and grainyhead (21–24).

Human LSF is a 502-amino acid protein with a molecular weight of ~57 kDa (24). It consists of two functional domains. The N-terminal domain is a DNA interaction region located between amino acids 67 and 260, and is similar in structure to p53/p63/p73 DNA binding domain. The C-terminal region is responsible for oligomerization and contains tetramerization and dimerization domains. The tetramerization domain is located between amino acids 326 and 89, and is structurally similar to the sterile α-motif protein-protein interaction domain (24,25). The dimerization domain contains residues 448–502 and is structurally similar to the ubiquitin-like fold domain (24–26). Amino acid residues 189–239 mediate nuclear localization of LSF (24,27,28).

With the exception of its important role in regulation of viral and cellular promoters, including SV40, and HIV-1 promoters, including fibrinogen and α-globin, LSF is involved in numerous other important biological functions, such as regulation of the cell cycle, cell growth and development, DNA synthesis, cell survival and Alzheimer’s disease. Moreover, LSF also functions as an anti-apoptotic factor (24,29,30).

The aim of the present study was to analyze TSG101 and LSF protein expression during cervical cancer development.

## Materials and methods

### Clinical samples

Samples were collected from 116 patients (median age, 49 years; range, 23–61 years) undergoing gynecological surgical procedures at the Department of Obstetrics and Pathology of Pregnancy, Medical University (Lublin, Poland). The study group consisted of 29 HPV-positive cervical samples (carcinoma colli uteri/squamous cell carcinoma), 30 HPV-positive high-grade squamous intraepithelial lesion (HSIL) samples, 28 HPV-positive low-grade squamous intraepithelial lesion (LSIL) samples and 29 histopathologically normal, HPV-negative cervical tissues obtained from women undergoing treatment for reasons other than cervical cancer. The study was approved by the Ethics Committee of the Medical University of Lublin. For cancer and neoplastic localization, all specimens initially underwent hematoxylin and eosin staining followed by a pathological review. Cervical sections comprising ≥70% cancer cells were used as cancer samples. The tissue samples were frozen immediately in liquid nitrogen and stored at −80°C until further analysis. Patients provided written informed consent.

### Isolation of DNA

Total DNA was isolated from study cells using a QIAmp DNA Midi kit (Qiagen, Hilden, Germany) according to the manufacturer’s instructions.

### Identification of HPV DNA

HPV DNA was identified by polymerase chain reaction (PCR) amplification of the HPV gene sequence using isolated DNA and primers: MY09, MY11 (31) and LC1, and LC2 (32), complementary to the genome sequence of the majority of common types of HPV viruses, as described previously (31,32).

### RNA extraction/isolation

Total RNA was isolated from normal, dysplastic (LSIL and HSIL) and cancer tissues using an RNeasy Mini kit (Qiagen) following the manufuacturer’s instructions. DNA was removed by DNase treatment (RNase-Free DNase set, Qiagen).

### Quantitative PCR (qPCR) analysis

Total RNA (1 μg) was reverse transcribed to cDNA using the QuantiTect Reverse Transcription kit (Qiagen). PCR reactions were run under the following conditions: pre-denaturation at 95°C for 5 min, then 40 cycles at 95°C for 10 sec, 63.8°C for 20 sec, 72°C for 20 sec and a final extension at 72°C for 6 min. qPCR was performed on a Corbett Rotor-Gene 6000 (Corbett Life Science, Concorde, NSW, Australia).

PCR was performed using SYBR Green PCR master mix (Invitrogen Life Technologies, Carlsbad, CA, USA) and appropriate primers (forward, 5′TGGCCGACGAAGTGATTGAA 3′ and reverse, 5′GGGCAATGCAAGGACATCAC 3′ for the LSF gene). Total cDNA (2 μl) and primers were added to 8 μl Power SYBR Green PCR master mix.

Glyceraldehyde 3-phosphate dehydrogenase (GAPDH) was used as reference gene (33). All reactions for samples and housekeeping genes were run in triplicate.

### Immunohistochemical analysis of TSG101 and LSF expression

Immunohistochemical analysis was prepared using a LSAB System-HRP visualization kit (K0679; Dako, Carpinteria, CA, USA), mouse monoclonal anti-TSG101 antibody (Santa Cruz Biotechnology, Inc., Santa Cruz, CA, USA) and mouse monoclonal anti-LSF antibody (BD Transduction Laboratories™, San Jose, CA, USA). Antibodies were diluted in Dako antibody diluent with background-reducing component (S3022; Dako).

Immunohistochemical evaluations of TSG101 and LSF expression were performed independently by two pathomorphologists. The cells were counted on an Axiophote (Opton) fluorescence microscope (Carl Zeiss, Oberkochen, Germany) with ×200 magnification on a field of 16 squares (4×4 squares), which corresponded to an area of 0.25 mm^2^ (0.5×0.5 mm).

Positive staining was scored according to the percentage of cells with positive staining and staining intensity. Measurement of immunoreactive cells was performed using Cell-2 software, version 4.1 (University of Medical Sciences, Poznan, Poland). The quantitative method is based on the analysis of the color distribution and the optical density. The software identifies cells with a greater optical density than the background and, on the basis of the color ratio, classifies cells as immunoreactive. To determine the percentage of positive cells in the sections, the counts of immunopositive cells were divided by the total cell count. For each case, a minimum of 5,000 total cells were counted in a single section.

### Statistical analysis

Data were analyzed with Statistica software, version 6.1. (Statsoft, Krakow, Poland). Statistical significance was analyzed using Kruskal-Wallis with post-hoc Dunn’s test (immunohistochemistry) and one-way analysis of variance with Tukey’s post hoc test (qPCR). The difference was considered to be statistically significant when P-values were <0.05.

### Bioinformatics analysis

The localization and frequency of LSF binding sites in the *TSG101* promoter sequence were analyzed using Cister software (Zlab gene regulation tools, Boston Univeristy, Boston, MA, USA) as described previously (1).

## Results

### Bioinformatics analysis of LSF binding site frequency in the TSG101 promoter sequence

Analysis using Cister software distinguished 14 binding sites for LSF transcription factor in the *TSG101* promoter sequence (Table I).

### Detection of HPV viruses in clinical samples

Prior to molecular analysis, clinical samples were screened for the presence of HPV viruses. Only HPV-positive HSIL, LSIL and cancer cells, and the HPV-negative control, were used in these studies (Table II).

### Immunohistochemical analysis of TSG101 expression

**Figure 1:**
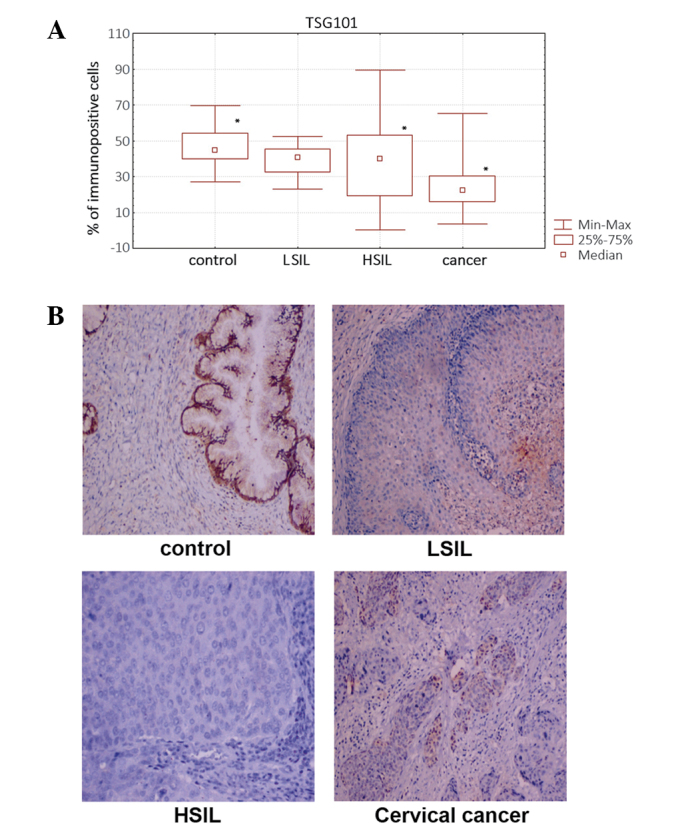
(A) Quantitative and (B) qualitative analysis of immunohistochemical staining for TSG101 (magnification, ×200). ^*^P<0.05 compared with non-tumor (HPV-negative) cells. HSIL, high-grade squamous intraepithelial lesion; LSIL, low-grade squamous intraepithelial lesion.

Quantitive immunohistochemical analysis based on the percentage of TSG101-immunopositive cells revealed a significantly (P<0.05) lower percentage of TSG101-immunopositive cells in cervical cancer and HSIL samples compared with that in non-tumor (HPV-negative) cells (Fig. 1A). The TSG101 protein showed mainly nuclear localization (Fig. 1B).

### Analysis of LSF mRNA and protein level

**Figure 2:**
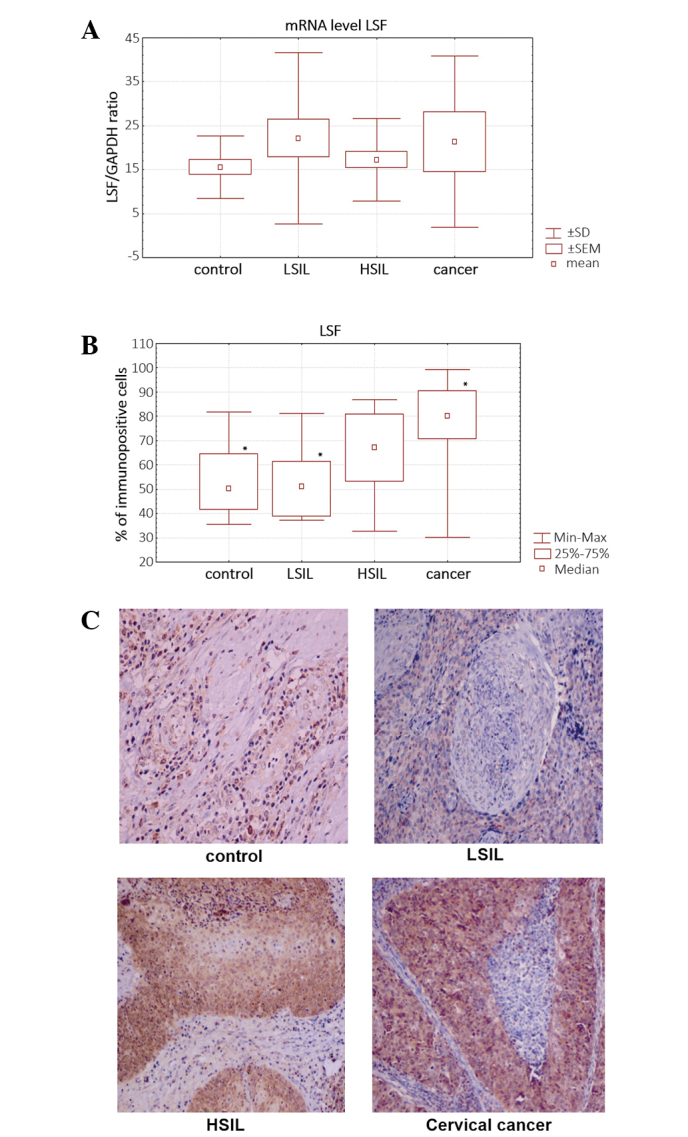
*LSF* mRNA level in HPV-negative non-tumor controls and LSIL, HSIL and HPV-positive cervical cancer epithelial cells. The *LSF* mRNA levels were determined by quantitative polymerase chain reaction analysis of cDNA. (A) Copy number of *LSF* transcripts was normalized to GAPDH expression level. (B) Quantitative and (C) qualitative analysis of immunohistochemical staining for LSF (magnification ×200). ^*^P<0.05 compared with non-tumor (HPV-negative) cells. LSF, late SV40 factor; HPV, human papillomavirus; HSIL, high-grade squamous intraepithelial lesion; LSIL, low-grade squamous intraepithelial lesion, GAPDH, glyceraldehyde 3-phosphate dehydrogenase.

qPCR analysis showed an increased level of *LSF* mRNA in dysplastic (LSIL and HSIL) and cervical cancer cells. *LSF* mRNA level was upregulated to 42.43% in HSIL samples, 30.6% in LSIL samples and 37.54% in cancer samples, compared with that in non-tumor HPV-negative samples (Fig. 2A). The differences in LSF expression were not statistically significant (P>0.05).

Quantitive immunohistochemical analysis based on the percentage of LSF-immunopositive cells confirmed the increased expression of LSF in cervical cancer cells.

The percentage of LSF-immunostained cells was significantly higher in cervical cancer and LSIL samples compared with that in HPV-negative non-tumor controls (P<0.05) (Fig. 2B). LSF was observed mainly in the nucleus (Fig. 2C).

## Discussion

In addition to HPV viruses, numerous factors, such as oncogenes and tumor suppressor genes are involved in cervical cancer development.

Our previous results demonstrated a decreased expression of TSG101 in cervical cancer cells (1). In the present study, we confirmed TSG101 protein downregulation during cervical cancer development using immunohistochemical analysis.

*TSG101* is constitutively expressed in a number of human tissues (34). However, upregulation of *TSG101* was found in thyroid papillary carcinomas, and breast, ovarian and gastrointestinal tumors, while downregulation of *TSG101* was observed in endometrial and cervical cancers (1,35,36).

Our bioinformatics analysis showed that one of the factors that may bind with the *TSG101* promoter and regulate its expression is LSF.

qPCR and immunohistochemical analysis revealed high expression levels of LSF in cervical cancer cells compared with non-cancer samples. These results suggest that LSF is important in cervical tumorigenesis and that LSF mRNA levels generally do not fluctuate. LSF mRNA has been suggested to be a normalization control in gene expression profiling (37). LSF is also expressed ubiquitously in cell lines and in the developing mouse, and protein levels are unaltered during cell cycle progression (38–40).

LSF is a transcription factor involved in the regulation of a variety of viral and cellular promoters. It acts as a transcription activator and repressor (24). LSF stimulates transcription of the SV40 late promoter (18). It also binds to the sequence within the HIV-1 long terminal repeat (LTR) initiation region and recruits YY1 and histone deacetylase 1 to the LTR, inhibiting transcription and thereby contributing to HIV persistence within resting CD4 T cells (41). The cellular factor YY1 also plays a critical role in tumorigenesis and HPV infection, as a positive and negative regulator of cellular and viral gene expression. The YY1-mediated downregulation of HPV transcription, as well as other promoters, act together with LSF (42).

High expression of LSF in cervical cancer HPV-positive cells suggests that this protein may be involved in downregulation of the *TSG101* gene promoter and HPV-dependent cervical carcinogenesis. Fan *et al* identified LSF as a downstream mediator of Notch1 signaling and showed that LSF mediates, at least in part, Notch-1-induced carcinogenesis (43). Notch genes encode heterodimeric transmembrane receptors, which play a critical role in maintaining the balance between cell proliferation, differentiation and apoptosis. Aberrant Notch signaling may contribute to cervical carcinogenesis (44), head and neck cancer (45), lung cancer (46), colon cancer (47), acute myeloid leukemia (48) and diffuse large B-cell lymphoma (30). LSF was also significantly upregulated in hepatocellular carcinoma compared with non-cancer samples (43). In liver cancer, its expression is strongly correlated with tumor grade and aggressiveness (49). Microarray studies revealed that LSF modulated the expression of specific genes involved in regulating invasion, angiogenesis, chemoresistance and senescence (24). It has been suggested that LSF may function as an oncogene in hepatocarcinogenesis (29,30,50). Osteopontin, matrix metalloproteinase 9, c-Met and complement factor H have been identified as a proteins directly regulated by LSF and involved in hepatocarcinogenesis (24).

A major cellular target of LSF is the thymidylate synthase gene, which encodes the enzyme involved in the production of dTTP, required for DNA synthesis. Deregulated LSF expression may facilitate entry into the G1/S phase of the cell cycle, promote DNA synthesis, stimulate transformation and facilitate cancer cell survival. Inhibition of LSF results in either apoptosis during S phase or cell cycle arrest at the G1/S transition (29,30,50).

The role of LSF as a mediator in cervical cancer development must be confirmed in future studies.

## Figures and Tables

**Table I:** Position and probability of LSF transcription factor binding to TSG101 promoter sequence according to Cister software.

Position	Strand	Sequence	Probablility
3262–3276	+	agtggcttacgcctg	0.56
5238–5252	−	ccggcccagccaagc	0.49
1158–1172	−	ccactgcactccagc	0.48
3398–3412	+	ggtggtgggcacctg	0.31
3293–3307	+	gcaggctgaggcggg	0.27
1333–1347	−	ctactgcactccagc	0.24
5174–5188	+	gctgcgacgcgctcg	0.21
1125–1139	+	ggtaggtggagcttg	0.19
5064–5078	−	ctggggcagcccagc	0.17
5233–5247	−	ccgtcccggcccagc	0.14
5053–5067	+	tgtgggacggtctgg	0.13
3493–3507	−	ctatcgcactccagc	0.12
6654–6668	−	caggcgtgagccacc	0.12
1063–1077	+	ggtggcaggtgcctg	0.10

LSF, late SV40 factor; TSG101, tumor susceptibility gene 101.

**Table II:** Characteristics of the studied group.

Clinical characteristics	Control	HSIL	LSIL	Cervical cancer
HPV detection	−	+	+	+
Number of patients	29	30	28	29

HSIL, high-grade squamous intraepithelial lesion; LSIL, low-grade squamous intraepithelial lesion; HPV, human papillomavirus.
